# 2-{(*E*)-[4-(Di­phenyl­amino)­phen­yl]imino­meth­yl}phenol

**DOI:** 10.1107/S1600536814003195

**Published:** 2014-02-15

**Authors:** Jiang Chen, Zhe-Peng Jin, Bing-Fei Gao, Jian-Hua Yu, Jie-Ying Wu

**Affiliations:** a Department of Chemistry, Bengbu Medical College, Bengbu 233030, People’s Republic of China; b Department of Chemistry, Anhui University, Hefei 230039, People’s Republic of China; c Key Laboratory of Functional Inorganic Materials, Chemistry, Hefei 230039, People’s Republic of China

## Abstract

The asymmetric unit of the title Schiff base molecule, C_25_H_20_N_2_O, contains two independent mol­ecules. In each mol­ecule, the C=N bond is in an *E* conformation. The most significant difference between the two mol­ecules is seen for the dihedral angles between the meth­oxy-substituted benzene ring and the two phenyl rings, which are 85.5 (1) and 82.3 (1)° in the first mol­ecule, and 49.0 (1) and 40.4 (1)° in the second. This conformational difference is reflected in the central C=N—C C torsion angle, which is 28.7 (2)° in the first mol­ecule and −29.8 (3)° in the other. In each mol­ecule, there is an intra­molecular O—H⋯N hydrogen bond.

## Related literature   

For related structures, see: Damous *et al.* (2013 ▶); Zheng (2013 ▶).
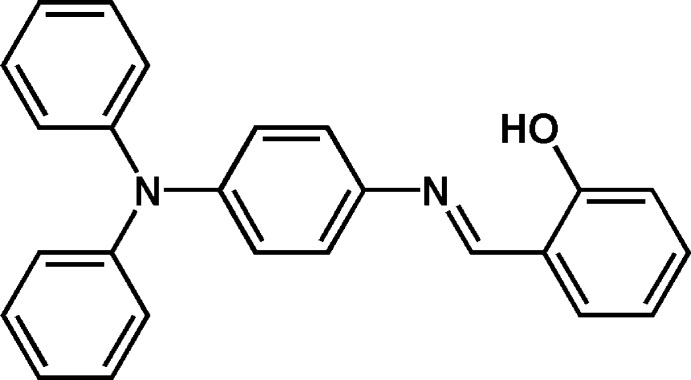


## Experimental   

### 

#### Crystal data  

C_25_H_20_N_2_O
*M* *_r_* = 364.43
Monoclinic, 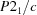

*a* = 18.4128 (4) Å
*b* = 21.2523 (4) Å
*c* = 10.2080 (2) Å
β = 99.834 (1)°
*V* = 3935.84 (14) Å^3^
*Z* = 8
Mo *K*α radiation
μ = 0.08 mm^−1^
*T* = 298 K
0.30 × 0.20 × 0.20 mm

#### Data collection  

Bruker SMART CCD diffractometer
Absorption correction: multi-scan (*SADABS*; Sheldrick, 1996 ▶) *T*_min_ = 0.978, *T*_max_ = 0.985
65034 measured reflections
8961 independent reflections
6499 reflections with *I* > 2σ(*I*)
*R*_int_ = 0.029

#### Refinement  

*R*[*F* ^2^ > 2σ(*F* ^2^)] = 0.049
*wR*(*F* ^2^) = 0.181
*S* = 1.09
8961 reflections
507 parameters
H-atom parameters constrained
Δρ_max_ = 0.13 e Å^−3^
Δρ_min_ = −0.18 e Å^−3^

### 

Data collection: *SMART* (Bruker, 2002 ▶); cell refinement: *SAINT* (Bruker, 2002 ▶); data reduction: *SAINT*; program(s) used to solve structure: *SHELXS97* (Sheldrick, 2008 ▶); program(s) used to refine structure: *SHELXL97* (Sheldrick, 2008 ▶); molecular graphics: *SHELXTL* (Sheldrick, 2008 ▶); software used to prepare material for publication: *SHELXTL*.

## Supplementary Material

Crystal structure: contains datablock(s) I, Global. DOI: 10.1107/S1600536814003195/lh5685sup1.cif

Structure factors: contains datablock(s) I. DOI: 10.1107/S1600536814003195/lh5685Isup2.hkl

Supporting information file. DOI: 10.1107/S1600536814003195/lh5685Isup3.cml

CCDC reference:

Additional supporting information:  crystallographic information; 3D view; checkCIF report

## Figures and Tables

**Table 1:** Hydrogen-bond geometry (Å, °)

*D*—H⋯*A*	*D*—H	H⋯*A*	*D*⋯*A*	*D*—H⋯*A*
O1—H1⋯N2	0.82	1.88	2.609 (2)	147
O2—H2⋯N4	0.82	1.86	2.590 (2)	148

**Fig. 1.**
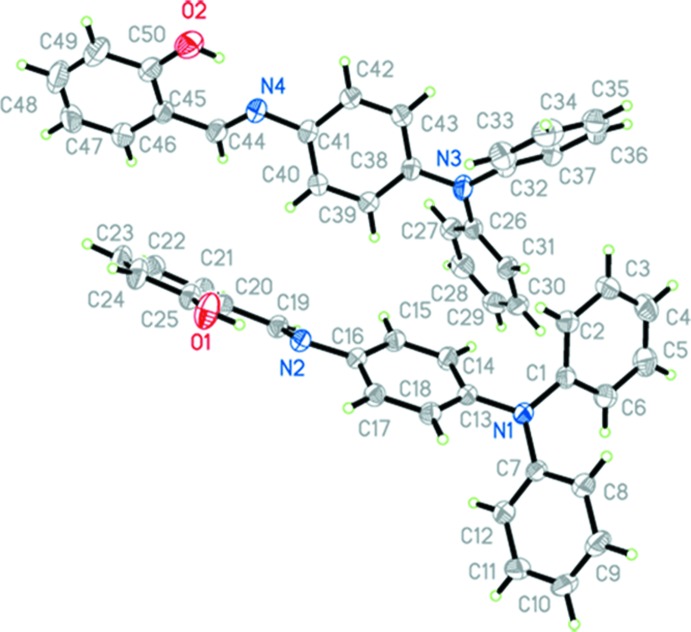
The molecular structure of (I). Displacement ellipsoids are drawn at the 30% probability level.

## supplementary crystallographic information

### 1. Comment

Schiff bases are considered important compounds because of their wide range of biological activities, and also because of their use as ligands in conjunction with transition metals (Damous *et al.*, 2013). Schiff bases derived from salicyladehyde and methylaniline with various substituents have exhibited potential application in pharmaceutical fields for their antitumor, antimicrobial and antiviral activities (Zheng *et al.*, 2013). Herein, with we report the crystal structure of the title compound (I).

In (I) (Fig.1), the Schiff base moiety shows an E configuration about the C16═N2 and C19═C20 bonds. The most significant difference between the two molecules are the dihedral angles between the methoxy-substituted benzene ring and the two phenyl rings, which are 85.5 (1)° [C20-C25/C1-C6] and 82.3 (1)° [C20-C25/C7-C12] in one molecule and 49.0 (1)° [C40-C50C26-C31] and 40.4 (1)° [C45-C50/C32-C37] in the other. This conformational difference is reflected in the central C═N—C≐C torsion angle which is 28.7 (2)° in one molecule [C19-N2-C16-C15] and -29.8 (3)° [C44-N4-C41-C40] in the other. The bond distances of the two independent molecules are the same within experimental error.

### 2. Experimental

A solution of *N*^1^,*N*^1^-diphenylbenzene-1,4-diamine (13.00 g, 50 ammol) in 30 ml of ethanol was mixed with 2-hydroxybenzaldehyde (9.15 g, 75 mmol) in ethanol 5 ml. The mixture was refluxed for 4h under cooling to room temperature, the soild was filtrated and recrystallized from ethnol to give X-ray quality crystals. Yield: 85%. ^1^H NMR (400 MHz, d^6^-(CD_3_)_2_CO) 6.99 (t, 2H), 7.12 (m, 8H), 7.36 (t, 4H), 7.41 (d, 3H), 7.59 (d, 1H), 8.93(d, 1H), 13.28 (s, 1H).

### 3. Refinement

All hydrogen atoms were placed in geometrically idealized positions and constrained to ride on their parent atoms, with C—H = 0.93 Å, O—H = 0.82Å and *U*_iso_(H) = 1.2*U*_eq_(C) or 1.5*U*_eq_(O).

### Crystal data

**Table:**

C_25_H_20_N_2_O	*F*(000) = 1536
*M**_r_* = 364.43	*D*_x_ = 1.230 Mg m^−^^3^
Monoclinic, *P*2_1_/*c*	Mo *K*α radiation, λ = 0.71073 Å
Hall symbol: -P 2ybc	Cell parameters from 9854 reflections
*a* = 18.4128 (4) Å	θ = 2.2–24.7°
*b* = 21.2523 (4) Å	µ = 0.08 mm^−^^1^
*c* = 10.2080 (2) Å	*T* = 298 K
β = 99.834 (1)°	Block, yellow
*V* = 3935.84 (14) Å^3^	0.30 × 0.20 × 0.20 mm
*Z* = 8	

### Data collection

**Table:**

Bruker SMART CCD diffractometer	8961 independent reflections
Radiation source: fine-focus sealed tube	6499 reflections with *I* > 2σ(*I*)
Graphite monochromator	*R*_int_ = 0.029
φ and ω scans	θ_max_ = 27.4°, θ_min_ = 2.2°
Absorption correction: multi-scan (*SADABS*; Sheldrick, 1996)	*h* = −23→18
*T*_min_ = 0.978, *T*_max_ = 0.985	*k* = −27→23
65034 measured reflections	*l* = −13→13

### Refinement

**Table:**

Refinement on *F*^2^	Primary atom site location: structure-invariant direct methods
Least-squares matrix: full	Secondary atom site location: difference Fourier map
*R*[*F*^2^ > 2σ(*F*^2^)] = 0.049	Hydrogen site location: inferred from neighbouring sites
*wR*(*F*^2^) = 0.181	H-atom parameters constrained
*S* = 1.09	*w* = 1/[σ^2^(*F*_o_^2^) + (0.1*P*)^2^ + 0.5243*P*] where *P* = (*F*_o_^2^ + 2*F*_c_^2^)/3
8961 reflections	(Δ/σ)_max_ = 0.001
507 parameters	Δρ_max_ = 0.13 e Å^−^^3^
0 restraints	Δρ_min_ = −0.18 e Å^−^^3^

### Special details

**Table:**

Geometry. All e.s.d.'s (except the e.s.d. in the dihedral angle between two l.s. planes) are estimated using the full covariance matrix. The cell e.s.d.'s are taken into account individually in the estimation of e.s.d.'s in distances, angles and torsion angles; correlations between e.s.d.'s in cell parameters are only used when they are defined by crystal symmetry. An approximate (isotropic) treatment of cell e.s.d.'s is used for estimating e.s.d.'s involving l.s. planes.
Refinement. Refinement of *F*^2^ against ALL reflections. The weighted *R*-factor *wR* and goodness of fit *S* are based on *F*^2^, conventional *R*-factors *R* are based on *F*, with *F* set to zero for negative *F*^2^. The threshold expression of *F*^2^ > σ(*F*^2^) is used only for calculating *R*-factors(gt) *etc*. and is not relevant to the choice of reflections for refinement. *R*-factors based on *F*^2^ are statistically about twice as large as those based on *F*, and *R*- factors based on ALL data will be even larger.

### Fractional atomic coordinates and isotropic or equivalent isotropic displacement parameters (Å^2^)

**Table:**

	*x*	*y*	*z*	*U*_iso_*/*U*_eq_	
N2	0.65015 (7)	0.18511 (7)	0.70724 (14)	0.0475 (3)	
O1	0.52431 (8)	0.12577 (8)	0.67606 (13)	0.0767 (4)	
H1	0.5657	0.1359	0.7130	0.115*	
C35	0.71752 (14)	0.54098 (15)	1.1730 (2)	0.0880 (8)	
H35	0.7229	0.5554	1.2602	0.106*	
N4	0.41701 (8)	0.39242 (7)	0.52112 (15)	0.0553 (4)	
N1	0.91196 (7)	0.25341 (7)	1.04786 (15)	0.0546 (4)	
O2	0.28230 (8)	0.36766 (9)	0.54581 (15)	0.0775 (4)	
H2	0.3237	0.3829	0.5651	0.116*	
N3	0.69482 (8)	0.47621 (8)	0.77970 (15)	0.0589 (4)	
C16	0.71657 (8)	0.20425 (7)	0.78898 (16)	0.0440 (3)	
C13	0.84654 (8)	0.23704 (7)	0.96105 (16)	0.0455 (4)	
C20	0.56131 (8)	0.19141 (8)	0.50738 (16)	0.0462 (4)	
C7	0.96755 (8)	0.20695 (8)	1.08522 (17)	0.0463 (4)	
C19	0.63139 (9)	0.20661 (8)	0.58888 (17)	0.0483 (4)	
H19	0.6639	0.2328	0.5543	0.058*	
C1	0.92484 (9)	0.31669 (7)	1.09053 (16)	0.0467 (4)	
C32	0.70191 (10)	0.49776 (9)	0.91339 (18)	0.0550 (4)	
C45	0.33276 (10)	0.34029 (8)	0.35242 (19)	0.0557 (4)	
C15	0.74933 (9)	0.26249 (8)	0.77910 (17)	0.0518 (4)	
H15	0.7277	0.2911	0.7152	0.062*	
C39	0.62060 (9)	0.40697 (8)	0.61860 (18)	0.0537 (4)	
H39	0.6634	0.3878	0.6016	0.064*	
C26	0.75879 (9)	0.47338 (8)	0.71989 (17)	0.0489 (4)	
C8	1.00166 (10)	0.20185 (9)	1.21639 (18)	0.0573 (4)	
H8	0.9875	0.2279	1.2807	0.069*	
C25	0.50993 (9)	0.15246 (9)	0.55383 (17)	0.0545 (4)	
C21	0.54279 (10)	0.21685 (9)	0.38024 (18)	0.0581 (4)	
H21	0.5768	0.2422	0.3475	0.070*	
C17	0.74919 (9)	0.16321 (8)	0.88735 (16)	0.0470 (4)	
H17	0.7276	0.1242	0.8961	0.056*	
C40	0.55297 (10)	0.38645 (9)	0.55257 (18)	0.0549 (4)	
H40	0.5507	0.3540	0.4909	0.066*	
C38	0.62562 (9)	0.45600 (8)	0.71015 (17)	0.0512 (4)	
C18	0.81303 (9)	0.17920 (8)	0.97237 (17)	0.0482 (4)	
H18	0.8338	0.1510	1.0378	0.058*	
C12	0.98805 (9)	0.16712 (8)	0.99112 (18)	0.0528 (4)	
H12	0.9648	0.1699	0.9030	0.063*	
C2	0.86863 (10)	0.35225 (8)	1.12765 (18)	0.0535 (4)	
H2A	0.8223	0.3345	1.1259	0.064*	
C11	1.04306 (10)	0.12317 (9)	1.0277 (2)	0.0616 (5)	
H11	1.0566	0.0963	0.9640	0.074*	
C14	0.81354 (9)	0.27842 (8)	0.86274 (18)	0.0533 (4)	
H14	0.8352	0.3173	0.8535	0.064*	
C6	0.99364 (10)	0.34357 (9)	1.0969 (2)	0.0612 (5)	
H6	1.0320	0.3203	1.0727	0.073*	
C44	0.40457 (10)	0.36586 (9)	0.40703 (19)	0.0580 (4)	
H44	0.4425	0.3629	0.3579	0.070*	
C41	0.48819 (9)	0.41398 (9)	0.57765 (17)	0.0524 (4)	
C50	0.27411 (10)	0.34239 (9)	0.4237 (2)	0.0612 (5)	
C9	1.05702 (11)	0.15786 (11)	1.2517 (2)	0.0680 (5)	
H9	1.0802	0.1548	1.3398	0.082*	
C27	0.75680 (10)	0.49235 (9)	0.58993 (19)	0.0574 (4)	
H27	0.7128	0.5063	0.5397	0.069*	
C43	0.56070 (10)	0.48410 (10)	0.73322 (19)	0.0606 (5)	
H43	0.5628	0.5177	0.7922	0.073*	
C31	0.82527 (10)	0.45261 (9)	0.7935 (2)	0.0595 (5)	
H31	0.8274	0.4396	0.8811	0.071*	
C30	0.88793 (11)	0.45130 (9)	0.7362 (3)	0.0705 (6)	
H30	0.9322	0.4375	0.7856	0.085*	
C28	0.82003 (12)	0.49053 (10)	0.5349 (2)	0.0696 (5)	
H28	0.8183	0.5033	0.4473	0.083*	
C46	0.32167 (12)	0.31212 (11)	0.2265 (2)	0.0757 (6)	
H46	0.3602	0.3108	0.1784	0.091*	
C29	0.88547 (12)	0.47023 (10)	0.6072 (3)	0.0732 (6)	
H29	0.9278	0.4693	0.5690	0.088*	
C3	0.88131 (12)	0.41414 (10)	1.1674 (2)	0.0673 (5)	
H3	0.8430	0.4380	1.1902	0.081*	
C22	0.47585 (12)	0.20549 (11)	0.3023 (2)	0.0676 (5)	
H22	0.4645	0.2230	0.2178	0.081*	
C42	0.49353 (10)	0.46248 (10)	0.6692 (2)	0.0605 (5)	
H42	0.4506	0.4809	0.6879	0.073*	
C24	0.44245 (10)	0.14073 (12)	0.4740 (2)	0.0704 (6)	
H24	0.4085	0.1144	0.5042	0.084*	
C10	1.07792 (10)	0.11884 (10)	1.1578 (2)	0.0666 (5)	
H10	1.1154	0.0896	1.1819	0.080*	
C23	0.42569 (11)	0.16797 (12)	0.3502 (2)	0.0726 (6)	
H23	0.3798	0.1608	0.2983	0.087*	
C37	0.74217 (11)	0.55151 (10)	0.9510 (2)	0.0651 (5)	
H37	0.7642	0.5734	0.8893	0.078*	
C33	0.66962 (13)	0.46571 (11)	1.0058 (2)	0.0724 (6)	
H33	0.6427	0.4293	0.9814	0.087*	
C4	0.94920 (14)	0.44041 (10)	1.1734 (3)	0.0803 (6)	
H4	0.9574	0.4820	1.2004	0.096*	
C36	0.74966 (13)	0.57282 (12)	1.0814 (2)	0.0815 (7)	
H36	0.7768	0.6091	1.1066	0.098*	
C5	1.00554 (13)	0.40513 (11)	1.1393 (3)	0.0801 (7)	
H5	1.0522	0.4228	1.1447	0.096*	
C49	0.20642 (12)	0.31691 (12)	0.3675 (3)	0.0850 (7)	
H49	0.1671	0.3186	0.4137	0.102*	
C47	0.25461 (16)	0.28644 (12)	0.1733 (3)	0.0941 (8)	
H47	0.2478	0.2673	0.0902	0.113*	
C34	0.67742 (15)	0.48791 (15)	1.1355 (2)	0.0883 (8)	
H34	0.6551	0.4664	1.1974	0.106*	
C48	0.19746 (14)	0.28941 (13)	0.2448 (3)	0.0976 (9)	
H48	0.1519	0.2724	0.2086	0.117*	

### Atomic displacement parameters (Å^2^)

**Table:**

	*U*^11^	*U*^22^	*U*^33^	*U*^12^	*U*^13^	*U*^23^
N2	0.0392 (7)	0.0522 (8)	0.0498 (8)	−0.0011 (6)	0.0035 (6)	−0.0029 (6)
O1	0.0643 (8)	0.1061 (12)	0.0574 (8)	−0.0312 (8)	0.0041 (6)	0.0169 (8)
C35	0.0838 (16)	0.117 (2)	0.0560 (12)	0.0424 (15)	−0.0093 (11)	−0.0196 (14)
N4	0.0454 (7)	0.0602 (9)	0.0582 (9)	−0.0063 (6)	0.0029 (6)	−0.0003 (7)
N1	0.0470 (7)	0.0417 (7)	0.0671 (9)	0.0048 (6)	−0.0129 (6)	−0.0055 (7)
O2	0.0627 (8)	0.0956 (12)	0.0762 (10)	0.0009 (8)	0.0177 (7)	0.0033 (8)
N3	0.0485 (8)	0.0746 (10)	0.0523 (8)	−0.0128 (7)	0.0052 (6)	−0.0119 (7)
C16	0.0364 (7)	0.0464 (8)	0.0484 (8)	0.0006 (6)	0.0043 (6)	−0.0021 (7)
C13	0.0415 (8)	0.0411 (8)	0.0505 (9)	0.0025 (6)	−0.0017 (6)	−0.0028 (7)
C20	0.0411 (8)	0.0505 (9)	0.0462 (8)	−0.0019 (6)	0.0054 (6)	−0.0053 (7)
C7	0.0381 (7)	0.0426 (8)	0.0556 (9)	0.0010 (6)	0.0006 (6)	0.0021 (7)
C19	0.0412 (8)	0.0530 (9)	0.0507 (9)	−0.0044 (7)	0.0079 (7)	−0.0017 (7)
C1	0.0483 (8)	0.0418 (8)	0.0469 (8)	−0.0017 (6)	−0.0012 (7)	−0.0014 (7)
C32	0.0509 (9)	0.0603 (10)	0.0511 (9)	0.0002 (8)	0.0010 (7)	−0.0055 (8)
C45	0.0487 (9)	0.0498 (10)	0.0647 (11)	−0.0001 (7)	−0.0010 (8)	−0.0008 (8)
C15	0.0522 (9)	0.0434 (9)	0.0548 (10)	0.0044 (7)	−0.0051 (7)	0.0049 (7)
C39	0.0464 (9)	0.0543 (10)	0.0596 (10)	0.0001 (7)	0.0067 (7)	−0.0062 (8)
C26	0.0464 (8)	0.0418 (8)	0.0571 (10)	−0.0062 (7)	0.0046 (7)	−0.0027 (7)
C8	0.0530 (9)	0.0629 (11)	0.0530 (10)	0.0075 (8)	0.0001 (8)	−0.0029 (8)
C25	0.0464 (9)	0.0667 (11)	0.0500 (9)	−0.0082 (8)	0.0074 (7)	−0.0037 (8)
C21	0.0576 (10)	0.0627 (11)	0.0527 (10)	−0.0075 (8)	0.0055 (8)	0.0019 (8)
C17	0.0459 (8)	0.0429 (8)	0.0511 (9)	−0.0043 (7)	0.0052 (7)	0.0030 (7)
C40	0.0517 (9)	0.0536 (10)	0.0576 (10)	−0.0033 (7)	0.0046 (8)	−0.0090 (8)
C38	0.0467 (9)	0.0546 (10)	0.0509 (9)	−0.0070 (7)	0.0045 (7)	−0.0031 (7)
C18	0.0498 (9)	0.0407 (8)	0.0507 (9)	0.0024 (7)	−0.0009 (7)	0.0056 (7)
C12	0.0527 (9)	0.0535 (10)	0.0501 (9)	0.0021 (7)	0.0032 (7)	0.0005 (8)
C2	0.0507 (9)	0.0490 (9)	0.0596 (10)	0.0017 (7)	0.0057 (7)	−0.0010 (8)
C11	0.0525 (10)	0.0597 (11)	0.0734 (12)	0.0094 (8)	0.0130 (9)	−0.0049 (9)
C14	0.0529 (9)	0.0380 (8)	0.0637 (10)	−0.0046 (7)	−0.0048 (8)	0.0051 (7)
C6	0.0512 (10)	0.0577 (11)	0.0738 (12)	−0.0053 (8)	0.0085 (9)	−0.0066 (9)
C44	0.0460 (9)	0.0674 (12)	0.0600 (11)	−0.0042 (8)	0.0076 (8)	−0.0023 (9)
C41	0.0450 (8)	0.0571 (10)	0.0537 (9)	−0.0073 (7)	0.0047 (7)	−0.0001 (8)
C50	0.0475 (9)	0.0579 (11)	0.0757 (13)	−0.0002 (8)	0.0039 (8)	0.0104 (10)
C9	0.0566 (10)	0.0810 (14)	0.0599 (11)	0.0139 (10)	−0.0090 (9)	0.0049 (10)
C27	0.0554 (10)	0.0523 (10)	0.0636 (11)	−0.0042 (8)	0.0074 (8)	0.0057 (8)
C43	0.0554 (10)	0.0631 (11)	0.0630 (11)	−0.0019 (8)	0.0092 (8)	−0.0172 (9)
C31	0.0565 (10)	0.0492 (10)	0.0684 (11)	−0.0023 (8)	−0.0019 (9)	0.0008 (8)
C30	0.0488 (10)	0.0493 (10)	0.1082 (18)	0.0018 (8)	−0.0011 (10)	−0.0123 (11)
C28	0.0740 (13)	0.0618 (12)	0.0772 (13)	−0.0143 (10)	0.0252 (11)	0.0013 (10)
C46	0.0661 (12)	0.0793 (14)	0.0757 (14)	0.0055 (10)	−0.0050 (10)	−0.0161 (12)
C29	0.0601 (12)	0.0543 (11)	0.1110 (19)	−0.0119 (9)	0.0310 (12)	−0.0178 (12)
C3	0.0758 (13)	0.0549 (11)	0.0682 (12)	0.0126 (9)	0.0038 (10)	−0.0106 (9)
C22	0.0696 (12)	0.0754 (13)	0.0517 (10)	−0.0030 (10)	−0.0071 (9)	0.0001 (9)
C42	0.0463 (9)	0.0679 (12)	0.0672 (11)	0.0000 (8)	0.0097 (8)	−0.0129 (9)
C24	0.0500 (10)	0.0926 (16)	0.0674 (13)	−0.0241 (10)	0.0064 (9)	−0.0085 (11)
C10	0.0463 (9)	0.0671 (12)	0.0825 (14)	0.0162 (8)	0.0002 (9)	0.0043 (11)
C23	0.0538 (10)	0.0930 (16)	0.0644 (12)	−0.0107 (10)	−0.0088 (9)	−0.0120 (11)
C37	0.0570 (10)	0.0642 (12)	0.0691 (12)	−0.0007 (9)	−0.0039 (9)	−0.0094 (10)
C33	0.0802 (14)	0.0755 (14)	0.0623 (12)	−0.0020 (11)	0.0149 (10)	0.0035 (10)
C4	0.0904 (16)	0.0478 (11)	0.0958 (17)	−0.0088 (11)	−0.0037 (13)	−0.0152 (11)
C36	0.0690 (13)	0.0803 (15)	0.0833 (16)	0.0147 (11)	−0.0211 (12)	−0.0294 (13)
C5	0.0685 (13)	0.0657 (13)	0.1025 (17)	−0.0241 (11)	0.0046 (12)	−0.0100 (12)
C49	0.0499 (11)	0.0879 (17)	0.113 (2)	−0.0064 (11)	0.0006 (12)	0.0179 (15)
C47	0.0868 (17)	0.0775 (16)	0.1023 (19)	0.0035 (13)	−0.0287 (15)	−0.0260 (14)
C34	0.0930 (17)	0.114 (2)	0.0592 (13)	0.0266 (16)	0.0154 (12)	0.0115 (14)
C48	0.0638 (14)	0.0815 (16)	0.132 (2)	−0.0167 (12)	−0.0281 (15)	0.0068 (16)

### Geometric parameters (Å, º)

**Table:**

N2—C19	1.283 (2)	C18—H18	0.9300
N2—C16	1.4173 (19)	C12—C11	1.382 (2)
O1—C25	1.355 (2)	C12—H12	0.9300
O1—H1	0.8200	C2—C3	1.385 (3)
C35—C34	1.366 (4)	C2—H2A	0.9300
C35—C36	1.368 (4)	C11—C10	1.376 (3)
C35—H35	0.9300	C11—H11	0.9300
N4—C44	1.279 (2)	C14—H14	0.9300
N4—C41	1.415 (2)	C6—C5	1.384 (3)
N1—C13	1.4115 (19)	C6—H6	0.9300
N1—C1	1.421 (2)	C44—H44	0.9300
N1—C7	1.427 (2)	C41—C42	1.384 (3)
O2—C50	1.342 (2)	C50—C49	1.390 (3)
O2—H2	0.8200	C9—C10	1.371 (3)
N3—C38	1.415 (2)	C9—H9	0.9300
N3—C26	1.418 (2)	C27—C28	1.377 (3)
N3—C32	1.424 (2)	C27—H27	0.9300
C16—C17	1.387 (2)	C43—C42	1.376 (3)
C16—C15	1.388 (2)	C43—H43	0.9300
C13—C18	1.389 (2)	C31—C30	1.380 (3)
C13—C14	1.393 (2)	C31—H31	0.9300
C20—C21	1.393 (2)	C30—C29	1.371 (3)
C20—C25	1.399 (2)	C30—H30	0.9300
C20—C19	1.447 (2)	C28—C29	1.371 (3)
C7—C12	1.380 (2)	C28—H28	0.9300
C7—C8	1.383 (2)	C46—C47	1.374 (3)
C19—H19	0.9300	C46—H46	0.9300
C1—C6	1.381 (2)	C29—H29	0.9300
C1—C2	1.385 (2)	C3—C4	1.361 (3)
C32—C33	1.378 (3)	C3—H3	0.9300
C32—C37	1.380 (3)	C22—C23	1.372 (3)
C45—C46	1.400 (3)	C22—H22	0.9300
C45—C50	1.402 (3)	C42—H42	0.9300
C45—C44	1.450 (2)	C24—C23	1.377 (3)
C15—C14	1.377 (2)	C24—H24	0.9300
C15—H15	0.9300	C10—H10	0.9300
C39—C40	1.381 (2)	C23—H23	0.9300
C39—C38	1.392 (2)	C37—C36	1.391 (3)
C39—H39	0.9300	C37—H37	0.9300
C26—C27	1.381 (2)	C33—C34	1.390 (3)
C26—C31	1.394 (2)	C33—H33	0.9300
C8—C9	1.385 (3)	C4—C5	1.372 (3)
C8—H8	0.9300	C4—H4	0.9300
C25—C24	1.387 (2)	C36—H36	0.9300
C21—C22	1.369 (3)	C5—H5	0.9300
C21—H21	0.9300	C49—C48	1.367 (4)
C17—C18	1.379 (2)	C49—H49	0.9300
C17—H17	0.9300	C47—C48	1.380 (4)
C40—C41	1.391 (3)	C47—H47	0.9300
C40—H40	0.9300	C34—H34	0.9300
C38—C43	1.392 (3)	C48—H48	0.9300
			
C19—N2—C16	121.25 (14)	C1—C6—H6	120.0
C25—O1—H1	109.5	C5—C6—H6	120.0
C34—C35—C36	119.5 (2)	N4—C44—C45	121.68 (17)
C34—C35—H35	120.2	N4—C44—H44	119.2
C36—C35—H35	120.2	C45—C44—H44	119.2
C44—N4—C41	121.57 (16)	C42—C41—C40	118.26 (15)
C13—N1—C1	120.37 (13)	C42—C41—N4	118.07 (16)
C13—N1—C7	119.58 (13)	C40—C41—N4	123.53 (16)
C1—N1—C7	119.90 (13)	O2—C50—C49	119.3 (2)
C50—O2—H2	109.5	O2—C50—C45	121.35 (16)
C38—N3—C26	120.95 (14)	C49—C50—C45	119.3 (2)
C38—N3—C32	120.38 (14)	C10—C9—C8	120.57 (18)
C26—N3—C32	118.64 (13)	C10—C9—H9	119.7
C17—C16—C15	118.26 (14)	C8—C9—H9	119.7
C17—C16—N2	117.98 (14)	C28—C27—C26	119.87 (18)
C15—C16—N2	123.72 (14)	C28—C27—H27	120.1
C18—C13—C14	118.24 (14)	C26—C27—H27	120.1
C18—C13—N1	120.63 (14)	C42—C43—C38	120.32 (17)
C14—C13—N1	121.13 (15)	C42—C43—H43	119.8
C21—C20—C25	118.23 (15)	C38—C43—H43	119.8
C21—C20—C19	120.08 (15)	C30—C31—C26	119.92 (19)
C25—C20—C19	121.68 (15)	C30—C31—H31	120.0
C12—C7—C8	119.53 (15)	C26—C31—H31	120.0
C12—C7—N1	120.49 (15)	C29—C30—C31	120.57 (19)
C8—C7—N1	119.98 (16)	C29—C30—H30	119.7
N2—C19—C20	122.16 (15)	C31—C30—H30	119.7
N2—C19—H19	118.9	C29—C28—C27	121.1 (2)
C20—C19—H19	118.9	C29—C28—H28	119.5
C6—C1—C2	118.92 (16)	C27—C28—H28	119.5
C6—C1—N1	120.64 (16)	C47—C46—C45	120.9 (2)
C2—C1—N1	120.44 (15)	C47—C46—H46	119.6
C33—C32—C37	119.53 (19)	C45—C46—H46	119.6
C33—C32—N3	121.15 (18)	C30—C29—C28	119.42 (19)
C37—C32—N3	119.32 (18)	C30—C29—H29	120.3
C46—C45—C50	119.02 (18)	C28—C29—H29	120.3
C46—C45—C44	119.56 (18)	C4—C3—C2	120.7 (2)
C50—C45—C44	121.42 (17)	C4—C3—H3	119.6
C14—C15—C16	120.79 (15)	C2—C3—H3	119.6
C14—C15—H15	119.6	C21—C22—C23	119.31 (19)
C16—C15—H15	119.6	C21—C22—H22	120.3
C40—C39—C38	120.99 (16)	C23—C22—H22	120.3
C40—C39—H39	119.5	C43—C42—C41	121.61 (17)
C38—C39—H39	119.5	C43—C42—H42	119.2
C27—C26—C31	119.14 (17)	C41—C42—H42	119.2
C27—C26—N3	120.91 (15)	C23—C24—C25	120.10 (18)
C31—C26—N3	119.94 (16)	C23—C24—H24	119.9
C7—C8—C9	119.82 (18)	C25—C24—H24	119.9
C7—C8—H8	120.1	C9—C10—C11	119.59 (17)
C9—C8—H8	120.1	C9—C10—H10	120.2
O1—C25—C24	118.70 (17)	C11—C10—H10	120.2
O1—C25—C20	121.50 (15)	C22—C23—C24	120.84 (18)
C24—C25—C20	119.80 (17)	C22—C23—H23	119.6
C22—C21—C20	121.70 (18)	C24—C23—H23	119.6
C22—C21—H21	119.2	C32—C37—C36	119.8 (2)
C20—C21—H21	119.2	C32—C37—H37	120.1
C18—C17—C16	121.19 (15)	C36—C37—H37	120.1
C18—C17—H17	119.4	C32—C33—C34	119.9 (2)
C16—C17—H17	119.4	C32—C33—H33	120.1
C39—C40—C41	120.48 (17)	C34—C33—H33	120.1
C39—C40—H40	119.8	C3—C4—C5	119.5 (2)
C41—C40—H40	119.8	C3—C4—H4	120.2
C43—C38—C39	118.32 (15)	C5—C4—H4	120.2
C43—C38—N3	120.70 (16)	C35—C36—C37	120.6 (2)
C39—C38—N3	120.98 (16)	C35—C36—H36	119.7
C17—C18—C13	120.57 (15)	C37—C36—H36	119.7
C17—C18—H18	119.7	C4—C5—C6	120.6 (2)
C13—C18—H18	119.7	C4—C5—H5	119.7
C7—C12—C11	120.09 (17)	C6—C5—H5	119.7
C7—C12—H12	120.0	C48—C49—C50	120.3 (2)
C11—C12—H12	120.0	C48—C49—H49	119.9
C3—C2—C1	120.12 (17)	C50—C49—H49	119.9
C3—C2—H2A	119.9	C46—C47—C48	119.2 (2)
C1—C2—H2A	119.9	C46—C47—H47	120.4
C10—C11—C12	120.39 (18)	C48—C47—H47	120.4
C10—C11—H11	119.8	C35—C34—C33	120.7 (3)
C12—C11—H11	119.8	C35—C34—H34	119.7
C15—C14—C13	120.93 (15)	C33—C34—H34	119.7
C15—C14—H14	119.5	C49—C48—C47	121.4 (2)
C13—C14—H14	119.5	C49—C48—H48	119.3
C1—C6—C5	120.06 (19)	C47—C48—H48	119.3
			
C19—N2—C16—C17	−153.60 (16)	N1—C13—C14—C15	−179.37 (16)
C19—N2—C16—C15	28.7 (2)	C2—C1—C6—C5	−0.3 (3)
C1—N1—C13—C18	−144.94 (17)	N1—C1—C6—C5	−179.68 (19)
C7—N1—C13—C18	39.5 (2)	C41—N4—C44—C45	176.17 (17)
C1—N1—C13—C14	34.5 (3)	C46—C45—C44—N4	179.45 (19)
C7—N1—C13—C14	−141.05 (17)	C50—C45—C44—N4	−1.4 (3)
C13—N1—C7—C12	44.6 (2)	C39—C40—C41—C42	0.5 (3)
C1—N1—C7—C12	−130.95 (18)	C39—C40—C41—N4	−175.25 (17)
C13—N1—C7—C8	−135.76 (18)	C44—N4—C41—C42	154.47 (19)
C1—N1—C7—C8	48.6 (2)	C44—N4—C41—C40	−29.8 (3)
C16—N2—C19—C20	−175.25 (15)	C46—C45—C50—O2	178.58 (19)
C21—C20—C19—N2	178.81 (17)	C44—C45—C50—O2	−0.6 (3)
C25—C20—C19—N2	0.3 (3)	C46—C45—C50—C49	−0.4 (3)
C13—N1—C1—C6	−137.97 (18)	C44—C45—C50—C49	−179.59 (19)
C7—N1—C1—C6	37.6 (2)	C7—C8—C9—C10	−0.6 (3)
C13—N1—C1—C2	42.6 (2)	C31—C26—C27—C28	0.0 (3)
C7—N1—C1—C2	−141.80 (17)	N3—C26—C27—C28	178.53 (17)
C38—N3—C32—C33	44.7 (3)	C39—C38—C43—C42	1.7 (3)
C26—N3—C32—C33	−133.12 (19)	N3—C38—C43—C42	−177.35 (18)
C38—N3—C32—C37	−135.65 (19)	C27—C26—C31—C30	0.1 (3)
C26—N3—C32—C37	46.5 (2)	N3—C26—C31—C30	−178.45 (16)
C17—C16—C15—C14	1.6 (3)	C26—C31—C30—C29	−0.1 (3)
N2—C16—C15—C14	179.25 (16)	C26—C27—C28—C29	−0.1 (3)
C38—N3—C26—C27	43.8 (2)	C50—C45—C46—C47	−0.4 (3)
C32—N3—C26—C27	−138.44 (18)	C44—C45—C46—C47	178.8 (2)
C38—N3—C26—C31	−137.73 (18)	C31—C30—C29—C28	0.0 (3)
C32—N3—C26—C31	40.1 (2)	C27—C28—C29—C30	0.1 (3)
C12—C7—C8—C9	1.4 (3)	C1—C2—C3—C4	−1.4 (3)
N1—C7—C8—C9	−178.22 (18)	C20—C21—C22—C23	−0.2 (3)
C21—C20—C25—O1	179.59 (18)	C38—C43—C42—C41	−2.1 (3)
C19—C20—C25—O1	−1.8 (3)	C40—C41—C42—C43	0.9 (3)
C21—C20—C25—C24	−0.6 (3)	N4—C41—C42—C43	176.91 (18)
C19—C20—C25—C24	177.94 (18)	O1—C25—C24—C23	178.9 (2)
C25—C20—C21—C22	1.2 (3)	C20—C25—C24—C23	−0.8 (3)
C19—C20—C21—C22	−177.42 (18)	C8—C9—C10—C11	−0.6 (3)
C15—C16—C17—C18	−0.8 (2)	C12—C11—C10—C9	0.9 (3)
N2—C16—C17—C18	−178.64 (15)	C21—C22—C23—C24	−1.3 (3)
C38—C39—C40—C41	−0.8 (3)	C25—C24—C23—C22	1.8 (4)
C40—C39—C38—C43	−0.3 (3)	C33—C32—C37—C36	−0.1 (3)
C40—C39—C38—N3	178.76 (17)	N3—C32—C37—C36	−179.76 (18)
C26—N3—C38—C43	−148.57 (18)	C37—C32—C33—C34	0.4 (3)
C32—N3—C38—C43	33.7 (3)	N3—C32—C33—C34	−179.92 (19)
C26—N3—C38—C39	32.4 (3)	C2—C3—C4—C5	0.2 (4)
C32—N3—C38—C39	−145.35 (18)	C34—C35—C36—C37	−0.3 (3)
C16—C17—C18—C13	−0.3 (3)	C32—C37—C36—C35	0.0 (3)
C14—C13—C18—C17	0.6 (3)	C3—C4—C5—C6	1.1 (4)
N1—C13—C18—C17	−179.89 (16)	C1—C6—C5—C4	−1.0 (4)
C8—C7—C12—C11	−1.0 (3)	O2—C50—C49—C48	−178.2 (2)
N1—C7—C12—C11	178.60 (16)	C45—C50—C49—C48	0.8 (3)
C6—C1—C2—C3	1.5 (3)	C45—C46—C47—C48	0.9 (4)
N1—C1—C2—C3	−179.12 (17)	C36—C35—C34—C33	0.6 (4)
C7—C12—C11—C10	−0.2 (3)	C32—C33—C34—C35	−0.7 (4)
C16—C15—C14—C13	−1.2 (3)	C50—C49—C48—C47	−0.3 (4)
C18—C13—C14—C15	0.1 (3)	C46—C47—C48—C49	−0.5 (4)

### Hydrogen-bond geometry (Å, º)

**Table:**

*D*—H···*A*	*D*—H	H···*A*	*D*···*A*	*D*—H···*A*
O1—H1···N2	0.82	1.88	2.609 (2)	147
O2—H2···N4	0.82	1.86	2.590 (2)	148
